# The Medico-Legal Import of the Opium Habit

**Published:** 1881-08

**Authors:** Ed. Levinstein, Herman Mynter


### Translations.

### The Medico-Legal Import of the Opium Habit— By Ed. Levinstein.

Translated from the German by Herman Mynter, M. D.

The author has found by the examination of morphium-eaters peculiar symptoms, not formerly described: difference in the size of the pupils, and disturbances of the accommodation; irregularity of the heart's action, and of respiration; loss of appetite, combined with feeling of intense hunger and thirst; cerebral symptoms, as uneasiness, fear, hallucinations, increased spinal reflex irritability, tremor of hands; albuminuria, impotence and amenorrhœa. Besides these symptoms he describes three forms of morphium fever; the first form, the morphium-intermittent fever, has the same clinical symptoms as a malarial intermittent fever, frost with following heat and perspiration in a tertian or quotidian type. The temperature of a morphium-intermittent fever reaches the same elevation as a malarial-intermittent fever, the intervals are as distinct, the spleen generally enlarged and often as much as in a severe malarial-intermittent fever. We may even find an erratic fever, with irregular attacks of frost with high temperature, heat and perspiration. Sometimes severe delirium may occur.

The second form of morphium fever is characterized by an almost daily (especially in the afternoon and evening) feeling of frost, increased heat and severe thirst, combined with moderate increase of temperature. These symptoms last generally for some hours, but may sometimes continue half a day.

The third form of morphium fever has symptoms like a typhoid fever, headache, ringing in the ears, dizziness. The patients feel weak and languid; keep their bed for three to six weeks; are unable to occupy themselves with anything; cannot read. The examination reveals a paresis of the accommodation, which is characteristic for this form. The temperature rarely increases more than 101.

All these symptoms of chronic morphium intoxication disappear instantly as soon as the use of morphine is discontinued. The irregularity of the eyes and of the digestion, the neuralgias and tremor disappear within eight days, the impotence in the third or fourth week; regular menstruation occurs in four to six weeks, even if amenorrhœa has lasted for years; the morphium-fever and attacks of intermittent fever, which may have lasted for months, disappear immediately, whether we discontinue the use of morphine suddenly or gradually.

The author has in former works declared his belief, that the sudden discontinuance is preferable to the gradual, which has the disadvantage, that it is very difficult, after weeks of treatment, to get rid of the last small doses, (may be only 1 centigram or even 5 milligram) and that the discontinuance of these last small doses is apt to produce the severe symptoms of abstinence, which, by sudden discontinuance, are overcome in four to five days.

To be sure, the sudden discontinuance produces violent symptoms; and weak and feeble patients, especially women, bear it with great difficulty, while patients with chronic, painful and incurable diseases cannot use it at all.

Some might say that it is unnecessary to try to cure the morphium habit in such patients, and in reality it is not advisable by patients, who have but a short time to live; on the other hand, it may be attempted with patients suffering from painful chronic diseases, which may last for years, as morphine, even in increasing doses, loses its power at last; the nervous system is satiated, and the morphine only intoxicates, but does not relieve the pain. If we, in such cases, discontinue its use for a time, the organism returns to its normal state, in which minimal doses of morphine have a narcotic action. In such cases the author recommends a modified treatment, and has used it with effect in phthisis, emphysema, diseases of heart and diabetes.

Before treatment is commenced, the patient is isolated for a few days to determine the daily amount of morphine he is in the habit of using. During these days he receives the amount of morphine he mentions as his habitual doses, subcutaneously; thereafter, the injections are discontinued at once. Very exceptionally a severe collapse will occur in the course of 24 hours after the last injection, and only if the nutrition of the patient is neglected.

At this time severe symptoms of abstinence will appear, from which a severe collapse may develop. As soon as a state of debility (intermittent pulse, slow and irregular respiration, colliquative diarrhœas, severe vomiting) threatens to appear, the perfect development of the same must be prevented. If the patient formerly was in the habit of using very large doses of morphine (1½—2 gram), one-thirtieth of the amount subcutaneously will be sufficient to produce, if not an agreeable, then an endurable condition.

By large doses (0.5 to 1,0) one-fifteenth, by small doses (less than 0,5) one-tenth of the amount subcutaneously will be sufficient. It is best to commence the cure in the evening. The patient has then very trifling symptoms during the first night, but they increase during the following day and reach their highest point in the evening. A subcutaneous injection of morphine will then produce a tolerable night. Next morning the symptoms of abstinence commence again, increase continually till evening; the patient gets then another, but smaller injection, respectively one-fortieth, one-twentieth, one-fifteenth of the habitual dosis. The same treatment with continually decreasing doses is used on the third, fourth and fifth day and so long, tilll the patient gets along with a dose of 0.03 to 0,01. The amount of morphine a patient with a chronic disease will have to use afterwards, can only approximatively be determined; the author believes that it will seldom be necessary to increase the last mentioned doses (0.1 to 0.3). The author has used the same modified treatment with very sensitive and weak patients. After 24 hours he gave by injection one-thirtieth, one-fifteenth, one-tenth of the former dose, according to its size, after 48 hours one-fortieth, one-twentieth, one-fifteenth; very seldom injections are necessary on the third and fourth day. We have learned by this modified treatment, that a small dose of morphine will be sufficient to prevent severe symptoms from developing, and will make the treatment endurable. The indications for the different forms of treatment are therefore the following:

Absolute and sudden discontinuance may be used by strong persons; the modified treatment with the intention to discontinue its use at last, by women and sensitive men; the modified treatment with the intention to diminish the habitual dose as much as possible by patients with chronic, incurable and painful diseases. Although the modified treatment is without danger, the author advices the same attention as by the sudden discontinuance.

The author mentions that there are morphine-eaters, who are unable to discontinue its use for a long time or always. They have been using large doses (1.5 to 2 gram) for a long time (10 to 15 years). They do not feel satisfied after the treatment is finished. They had regained their appetite and were able to sleep, looked healthy, but they felt sick. In the course of five to six months they lost their appetite again, became sleepless, looked miserable and commenced to decline in health, although the objective examination revealed nothing. Morphine has become a necessity in these rare cases, and they are easily cured by morphine in small doses, 5 to 10 milligram two to three times a day. Old persons may always safely discontinue its use for 6 months; persons in middle age, for one year.

The author has treated 110 morphine-eaters, of which 82 were men, 28 woman. The sex has no influence, but the social position and business of the male has great influence. Physicians furnish the largest proportion. Of the 110 morphine-eaters, 32 were physicians; eight, wives of physicians; one, a son of a doctor; two sisters of charity, two nurses, one midwife, one medical student; in all 47 persons, belonging to the medical profession. After them the military furnishes the largest proportion; eighteen being officers, and one the wife of an officer. Six were druggists, one the wife of a druggist, eleven merchants, five wives of merchants, four wives of officials, two unmarried ladies, etc. The youngest was 21 years, the oldest 65 years. Twenty men and women had become accustomed to the use of morphine on account of acute diseases, one man used it as antaphrodisiacum. 15 men and women took to its use on account of troubles in the house, etc.; 12 men became drunkards during its use. Of the 82 men 61 relapsed, of 28 women 10, of the 32 physicians 28. The easier the opportunity of getting and handling morphine, the more probable the relapse. It is almost impossible to prevent relapse in druggists. We must take great care not to give patients, who have been morphine-eaters, subcutaneous injections of morphine, when they complain of pain. The author has observed several relapses from this cause. Four physicians died from its use; three of them had gradually increased the dose, till they reached a fatal one. They were found dying in their beds with symptoms of acute morphine-poisoning. The fourth died during the cure of abstinence from an overdose of morphine he had procured (1/3 of his habitual dose) and which, at that state, was fatal.—*Berliner Klinische Wochenschrift.* 1880. No. 6.

